# Plant Flavonoids—Biosynthesis, Transport and Involvement in Stress Responses

**DOI:** 10.3390/ijms140714950

**Published:** 2013-07-17

**Authors:** Elisa Petrussa, Enrico Braidot, Marco Zancani, Carlo Peresson, Alberto Bertolini, Sonia Patui, Angelo Vianello

**Affiliations:** Department of Agricultural and Environmental Sciences, Unit of Plant Biology, University of Udine, via delle Scienze 91, Udine I-33100, Italy; E-Mails: elisa.petrussa@uniud.it (E.P.); enrico.braidot@uniud.it (E.B.); marco.zancani@uniud.it (M.Z.); carlo.peresson@uniud.it (C.P.); alberto.bertolini@uniud.it (A.B.); sonia.patui@uniud.it (S.P.)

**Keywords:** flavonoid transport, bilitranslocase, ABC transporters, secondary metabolites, biotic and abiotic stress

## Abstract

This paper aims at analysing the synthesis of flavonoids, their import and export in plant cell compartments, as well as their involvement in the response to stress, with particular reference to grapevine (*Vitis vinifera* L.). A multidrug and toxic compound extrusion (MATE) as well as ABC transporters have been demonstrated in the tonoplast of grape berry, where they perform a flavonoid transport. The involvement of a glutathione *S*-transferase (GST) gene has also been inferred. Recently, a putative flavonoid carrier, similar to mammalian bilitranslocase (BTL), has been identified in both grape berry skin and pulp. In skin the pattern of BTL expression increases from *véraison* to harvest, while in the pulp its expression reaches the maximum at the early ripening stage. Moreover, the presence of BTL in vascular bundles suggests its participation in long distance transport of flavonoids. In addition, the presence of a vesicular trafficking in plants responsible for flavonoid transport is discussed. Finally, the involvement of flavonoids in the response to stress is described.

## 1. Introduction

Flavonoids are a group of plant polyphenolic secondary metabolites showing a common three ring chemical structure (C_6_–C_3_–C_6_). The major classes of flavonoids are anthocyanins (red to purple pigments), flavonols (colourless to pale yellow pigments), flavanols (colourless pigments that become brown after oxidation), and proanthocyanidins (PAs) or condensed tannins. These compounds are widely distributed in different amounts, according to the plant species, organ, developmental stage and growth conditions [1]. They perform a wide range of functions, such as antioxidant activity, UV-light protection and defence against phytopathogens (e.g., isoflavonoids, which play the role of phytoalexins in legumes), legume nodulation, male fertility, visual signals and control of auxin transport [2]. In particular, isoflavonoid phytoalexins of legumes are synthesized through a branch of the phenylpropanoid pathway.

Flavonoids are also the major component of the soluble phenolics found in grapevine (*Vitis vinifera* L.) tissues, with the exception of the nonflavonoid hydroxycinnamates, which are the most common phenolics in grape mesocarp and, particularly, in white cultivars [3,4]. Among the most abundant classes of grape flavonoids, PAs and catechins (a class of flavanols) are located in both skin and seed, whereas flavonols and anthocyanins are accumulated mainly in thick-walled hypodermal cells of the skin [4,5]; anthocyanins are also present in the mesocarp of “teinturier” grapes.

In red grape, the monoglycoside forms of anthocyanins are typical end-products of the phenylpropanoid metabolism. Then, they may be subjected to further esterification with acetyl or coumaroyl groups, as well as substitution with hydroxyl or methyl groups [4,6], thus increasing stabilization and colour variation of the pigments. Such additions could sometimes be essential to allow binding by transporters because, as demonstrated by Zhao and co-workers [7], flavonoid glycosides esterified with malonate are the preferential substrates of multidrug and toxic compound extrusion protein (MATE). Pigment accumulation in the skin during berry ripening takes place from *véraison* to harvest, conferring the natural pigmentation to mature fruits [8,9]. At cellular level, flavonoids need to be properly delivered to and stored in distinct compartments, mainly vacuole [2,10] and cell wall [11–13], like many other secondary metabolites [2,10]. Despite a comprehensive understanding of the flavonoid biosynthetic pathway, information about the mechanisms of their transport across endomembranes and subsequent accumulation into different compartments is still limited [6]. It has been proposed that some transporters, using different mechanisms, could co-exist in plant cells and be responsible for sequestration of the flavonoid molecules (for reviews see [2,6,10,14–16]). However, the molecular basis of vacuolar uptake of flavonoids (in particular anthocyanins) in plant cells, including grapevine [17–19], has been examined mainly by genomic approaches [2].

This paper aims to examine three aspects of flavonoid metabolism: (i) the synthesis in plant cells; (ii) the translocation and trafficking in grapevine cells, in the frame of the transport mechanisms already described for other plant species; and (iii) their involvement in the response to stress in the grapevine.

## 2. Biosynthetic Pathway of Flavonoids in Plant Cells

Flavonoids (in particular anthocyanins and PAs) are synthesized along the general phenylpropanoid pathway by the activity of a cytosolic multienzyme complex, known also as flavonoid metabolon, loosely associated to the cytoplasmic face of the endoplasmic reticulum (ER). In particular, some of these enzymes belong to the cytochrome-P450 family and possess the ability to bind to membranes [20,21]. On the other hand, some of the enzymes involved in the biosynthetic pathway are loosely associated with membranes of different organelles, such as vacuole [22–25], plastids and nucleus [26–28]. In particular, plastids from grapevine show the presence of the chalcone synthase (CHS) and leucoanthocyanidin oxidase (LDOX), the latter being described also in the nucleus [26–28]. Such findings may suggest that a multi-branching distribution of the enzymes involved in flavonoid biosynthesis might correspond to a peculiar function during berry maturation.

**Figure 1:**
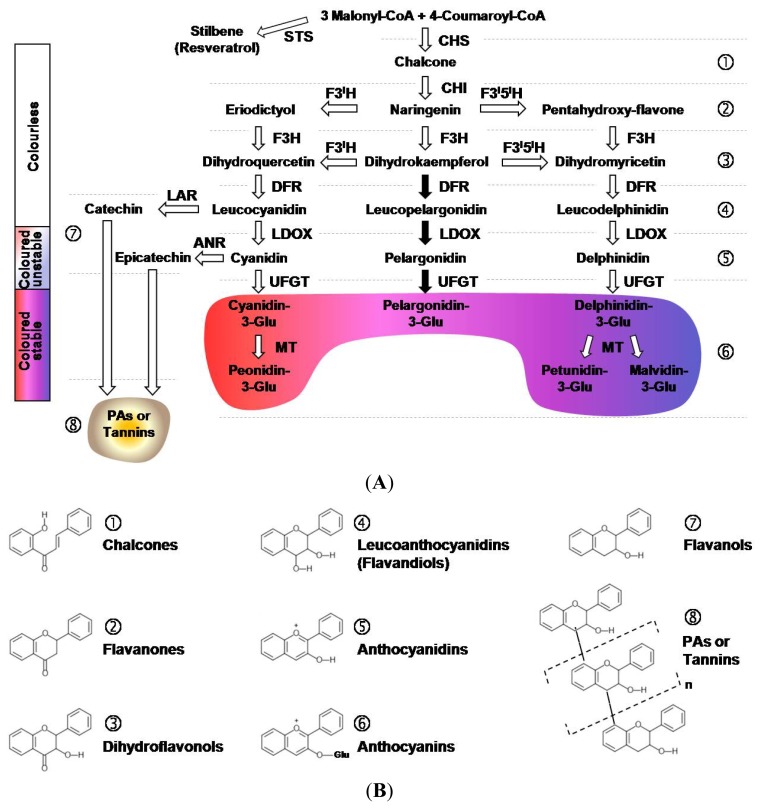
(**A**) Scheme of the flavonoid biosynthetic pathway in plant cells. Anthocyanins are synthesized by a multienzyme complex loosely associated to the endoplasmic reticulum (CHS, chalcone synthase; CHI, chalcone isomerase; F3H, flavanone 3-hydroxylase; F3′H, flavonoid 3′-hydroxylase; F3′5′H, flavonoid 3′,5′-hydroxylase; DFR, dihydroflavonol reductase; LDOX, leucoanthocyanidin oxidase; UFGT, UDP-glucose flavonoid 3-*O*-glucosyl transferase; MT, methyltransferase). Proanthocyanidins (PAs) synthesis branches off the anthocyanin pathway (LAR, leucoanthocyanidin reductase; ANR, anthocyanidin reductase; STS, stilbene synthase); the black arrows refer to biosynthetic steps missing in grapevine. Numbers next to the flavonoid groups are related to the chemical structures shown in (**B**). (**B**) Chemical structures of the major flavonoid groups.

The flavonoid biosynthetic pathway has largely been characterized (Figure 1), especially in *Arabidopsis thaliana* and *Zea mays*, but also in *V. vinifera* [5,8,29]. The upstream pathway consists in the formation of the core (the flavylium ion), the basic skeleton of all flavonoids, starting from three molecules of malonyl-CoA and one of 4-coumaroyl-CoA. CHS and chalcone isomerase (CHI) are the enzymes involved in the two-step condensation, producing a colourless flavanone named naringenin. The oxidation of the latter compound by flavanone 3-hydroxylase (F3H) yields the dihydrokaempferol (colourless dihydroflavonol) that subsequently can be hydroxylated on the 3′ or 5′ position of the B-ring, by flavonoid 3′-hydroxylase (F3′H) or flavonoid 3′,5′-hydroxylase (F3′5′H), producing, respectively, dihydroquercetin or dihydromyricetin. Naringenin may also be directly hydroxylated by F3′H or F3′5′H to deliver, respectively, eriodictyol and pentahydroxy-flavanone, which are again hydroxylated to dihydroquercetin and dihydromyricetin. The three dihydroflavonols thus synthesized are then converted to anthocyanidins (coloured but unstable pigments) by two reactions catalysed by dihydroflavonol reductase (DFR) and LDOX. The DFR converts dihydroquercetin, dihydrokaempferol and dihydromyricetin to leucocyanidin, leucopelargonidin and leucodelphinidin (colourless flavan-3,4-*cis*-diols), respectively. Subsequently, LDOX catalyses the oxidation of leucocyanidin, leucopelargonidin and leucodelphinidin to cyanidin (red-magenta anthocyanidin), pelargonidin (orange anthocyanidin) and delphinidin (purple-mauve anthocyanidin), respectively. All the colours above mentioned refer to a specific environmental condition, *i.e.*, when the anthocyanidins are in an acidic compartment. The last common step for the production of coloured and stable compounds (anthocyanins) involves the glycosylation of cyanidin, pelargonidin and delphinidin by the enzyme UDP-glucose:flavonoid 3-*O*-glucosyl transferase (UFGT). Finally, only cyanidin-3-glucoside and delphinidin-3-glucoside may be further methylated by methyltransferases (MTs), to be converted to peonidin-3-glucoside and petunidin- or malvidin-3-glucoside, respectively.

The synthesis of PAs branches off the anthocyanin pathway after the reduction of leucocyanidin (or cyanidin) to catechin (or epicatechin) by the enzymatic activity of a leucoanthocyanidin reductase (LAR), or anthocyanidin reductase (ANR) [30]. The subsequent steps take place in the vacuolar compartments, where the formation of PA polymers occurs by the addition of leucocyanidin molecules to the terminal unit of catechin or epicatechin, possibly catalysed by laccase-like polyphenol oxidases. However, the localization of these enzymes and their actual substrates are still controversial [31,32].

## 3. Mechanisms of Flavonoid Transport in Plant Cells

In the following section, recent advances on the models of flavonoid transport into vacuole/cell wall of different plant species, ascribed to a general membrane transporter-mediated transport (MTT), will be examined, including a novel membrane transporter initially found in carnation petals.

The establishment of a proton gradient between the cytosol and the vacuole (or the cell wall) by H^+^-ATPases (and H^+^-PPases in the tonoplast) has been proposed as the main driving force for the transport of some flavonoids and, in particular, anthocyanins into vacuole [33]. Once these compounds are in the vacuoles, the acidic pH inside the vacuolar compartment and the acylation of flavonoids are both necessary for the induction of a conformational modification, responsible for the appropriate trapping and retention of the metabolites [2,34].

Besides the well-known role in secondary metabolism and xenobiotic detoxification, ATP-binding cassette (ABC) transporters have also been claimed to play a role in sequestration of flavonoids into the vacuole [10,35–37]. These proteins are capable of coupling the hydrolysis of ATP to a direct translocation, through the membranes, of many substrates after their conjugation with glutathione (GSH), by a reaction catalysed by glutathione *S*-transferases (GST) [37–40]. ABC transporters are structurally characterized by two cytosolic nucleotide-binding sites, NBF1 and NBF2, each containing a Walker motif (A and B, respectively). Their activity is inhibited by vanadate, an inhibitor of P-ATPases, while is insensitive to bafilomycin, a specific inhibitor of V-ATPases [39,40]. ABC transporters are also able to transport flavonoid glycosides, glucuronides and glutathione conjugates to the vacuole by a directly energized (primary) mechanism [6,41]. However, it is noteworthy that there is no evidence about anthocyanin-GSH conjugate found in plant cells [2,37]. The involvement of a subfamily of the ABC transporters, the multidrug resistance-associated protein (MRP/ABCC)-type (also named glutathione *S*-conjugate pump), in the transport of glutathionylated anthocyanins has been previously suggested by mutant analysis in maize and petunia [42,43]. Such mutants, defective in GST, are unable to accumulate anthocyanins into vacuoles [44–46], suggesting that GST proteins could act just as flavonoid binding proteins. These authors have proposed that, on the basis of the preference of MRP/ABCC for glutathione conjugates (as substrates), the ABC transporters could be the major candidates for their translocation into the vacuole, or to export them through the plasma membrane. Similar results have been reported in carnation (*Dianthus caryophyllus*) [47] and *Arabidopsis* [48]. Finally, further evidence on the involvement of MRP in anthocyanin deposition has been directly provided by the identification of MRP/ABCC proteins in maize, where it is present in the tonoplast and is necessary for anthocyanin accumulation into the aleurone layer [42]. In a very recent paper, Francisco and coworkers [49] have shown that free GSH is specifically co-transported with anthocyanidin 3-*O*-glucosides into microsomes of yeast expressing grapevine ABCC1. By *in vitro* assays, neither structural alterations of the transported anthocyanins nor GSH-conjugated forms have been detected. Hence, these authors concluded that GSH conjugation is not an essential prerequisite for anthocyanin transport mediated by ABCC transporters.

Genomic studies with *Arabidopsis transparent testa* (*tt*) mutants, defective in flavonoid biosynthesis occurring in the seed endothelium cells, suggest that different types of transporters could be involved in flavonoid transport across tonoplast [2]. This suggestion comes from the finding that the mutant *tt12* exhibits pigment deficiency in the seed coat due to the lack of vacuolar deposition of PAs [1]. The TT12 protein shows similarity to MATE transporters, which are distinct from the ABC-type ones [2], and function as secondary transporters for the uptake of PAs [1,15,50]. The participation of MATE transporters in flavonoid vacuolar sequestration have also been described in tomato [51] and *Arabidopsis* [1,7], where up-regulation of flavonoid biosynthesis and transporter-like genes are induced by activation of regulatory factors [2,51].

Previous studies in leaves from barley mutants, defective in flavonoid biosynthesis, have demonstrated that saponarin (a glycosylated flavone) and its precursor are accumulated into the vacuole by a proton/flavonoid antiporter [52]. The activity of this secondary transporter, being insensitive to vanadate (an inhibitor of the ABC transporters), resembles that performed by MATE-type protein, which instead requires an established vacuolar electrochemical proton gradient.

In contrast to what shown in barley, the uptake of saponarin in *Arabidopsis* vacuoles exhibits a different pattern, since the transport is mediated by an ABC-transporter [53]. Indeed, saponarin in *Arabidopsis* does not represent an endogenous secondary metabolite and could be, hence, recognized as a potentially toxic xenobiotic compound by the plant itself. These results corroborate the hypothesis that the transport of the same flavonoid molecule could be mediated by different mechanisms in various plant species [14,35]. For this reason, the authors assumed that endogenous glycosylated flavonoids are taken up into the vacuole by an antiporter driven by secondary energization (H^+^ gradient), whereas non-specific/xenobiotic compounds are accumulated for their proper detoxification by a primary mechanism mediated by MRP/ABCC transporters [35,38,50]. This assumption is in conflict with the observations made in petunia and maize above reported [42,43].

Besides the mechanisms proposed already, a new carrier, putatively involved in the transport of flavonoids, has been found in epidermal tissues of carnation petals [54]. This protein is similar to mammalian bilitranslocase (BTL), a plasma membrane carrier localized in liver and gastric mucosa, where it mediates the uptake of the tetrapyrrolic pigment bilirubin and other organic ions, such as dietary anthocyanins and nicotinic acid [55,56]. The BTL-homologue in carnation possesses, similarly to the mammalian carrier, an apparent molecular mass of 38 kDa and is localized in both purified tonoplast and plasma membrane vesicles. Its activity is measured as electrogenic transport of bromosulfalein (BSP), a phthalein with a molecular structure similar to flavonoids. BSP uptake is dependent on an electrogenic gradient, is competitively inhibited by cyanidin-3-glucoside and by cyanidin (mainly non-competitively). Moreover, it has been found that the electrogenic BSP uptake in carnation petal microsomes is insensitive to GSH and is not stimulated by ATP, confirming that such a carrier does not belong to the ABC transporter family.

## 4. Genetic Regulation of Flavonoid Transport in Plant Cells

The modulation of expression of flavonoid biosynthetic genes is one of the best-known regulatory systems of plants. In particular, the transcription factors so far described in *Arabidopsis*, maize, petunia and grapevine are: (i) the bHLH transcription factors, belonging to multigenic families, structurally organized into basic-helix-loop-helix DNA-binding conserved motifs [57–59]; (ii) the MYB proteins (binding DNA as well) involved in the control of the biosynthesis of all classes of flavonoids—Most of them have two R repeats (R2R3-MYB proteins) consisting of three imperfect repeats, each containing 53 aminoacids organized in a helix-turn-helix structure [59–61]; (iii) the WD-repeat-containing proteins, built up by four or more copies of the WD (tryptophan-aspartate) repeats, a sequence motif approximately 31 amino acid long that encodes a structural repeat [59,62]. These transcription factors could interact as ternary complexes MYB-bHLH-WD40 (MBW) in the regulation of genes encoding enzymes involved in the final steps of flavonoid biosynthetic pathway [59]. The structural genes of the flavonoid biosynthetic pathway are independently regulated in relation to the different branches where they are present; e.g., phlobaphene, anthocyanin, PA or flavonol biosynthesis [59,63].

Despite the scarce information about the regulation of the expression of genes encoding for proteins related to flavonoid transport, few examples have been reported. In particular, in *Arabidopsis* it has been described that AtTT2, a protein belonging to the R2R3-MYB protein family, controls the flavonoid late metabolism in developing siliques. It also regulates the expression of *TT12* gene that codes for a putative transporter, likely involved in vacuolar sequestration of PA precursors [64]. Furthermore, in maize, *ZmMRP3* expression (an ABCC transporter protein related to anthocyanin transport) is regulated by the transcription factors R (bHLH family) and C1 (R2R3-MYB protein family) [42]. Indeed, some of the above described transcription factors are also responsible for the activation of structural genes indirectly involved in the final steps of flavonoid translocation through the vacuolar membrane, such as *BZ2* in maize, *AN9* in petunia and *TT19* in *Arabidopsis*, all encoding GSTs [37,65].

## 5. Transport Mediated by Vesicle Trafficking in Plant Cells

The abovementioned membrane transporter-mediated transport (MTT) probably involves the participation of ligandins, such as GST, as carriers of flavonoids to be transported. However, emerging evidence suggests also the participation of a membrane vesicle-mediated transport (MVT) [65–69], involving a coordinated trafficking of flavonoid-containing vesicles from synthesis sites to the accumulation targets, as proposed for the secretion of many compounds (e.g., proteins and polysaccharides) [50]. For these reasons, the most probable hypothesis suggested by this model is that these vesicles could release their content into the vacuole by a fusion with the tonoplast [70].

Vesicles involved in the transport of flavonoid-derived compounds have been found in maize cells, induced to accumulate anthocyanins [68], and in sorghum cells, challenged by fungal infection [71]. The vesicular-type transport of anthocyanins from ER to the vacuole could cooperate with AN9/BZ2-like GSTs and/or tonoplast transporters [42,43,45,72], since these enzymes could be responsible for the uploading of pigments into the vesicles. Nevertheless, this model does not explain how flavonoids are uploaded into the ER compartment. Concerning this question, it has been hypothesized that flavonoid uptake into ER lumen might be mediated by membrane translocators or ligandin similar to the ones described for the vacuole (e.g., TT12, a MATE transporter; and TT19, a GST) [2]. Then, similarly to other metabolites, the flavonoid allocation could occur through different parallel pathways, the details of which are still poorly understood. Microscopy analyses by Lin and co-workers [73] have shown that phytochemicals are transported by at least two distinct vesicle trafficking pathways, addressed either to cell wall or to vacuole. The first one is a *trans* Golgi network (TGN)-independent pathway, suggesting that it is different from the secretion pathway of most proteins. The second one leads to the vacuolar accumulation of the compounds in anthocyanic vacuolar inclusions (AVIs), dark red- to purple-pigmented spherical bodies, either encased or not by lipid membranes. Such structures have been described, sometimes with contradictory results on localisation and molecular composition, in plant cell suspension cultures of sweet potato [34], petals of lisianthus (*Eusthonia* sp.) [67], carnation flowers [11], *Arabidopsis* seedlings [74], as well as in more than 70 anthocyanin-producing species [11,75]. In some cells, AVIs are associated to insoluble proteinaceous matrices.

Consistent with ER-to-vacuole vesicular transport of anthocyanins mediated by a TGN-independent mechanism, Poustka and co-workers [65] have demonstrated that Brefeldin A, a Golgi-disturbing agent [76], has no effect on the accumulation of anthocyanins. However, vanadate, a fairly general inhibitor of ATPases and ABC transporters, induces a dramatic increase of anthocyanin-filled sub-vacuolar structures. These results indicate that *Arabidopsis* cells, accumulating high levels of anthocyanins, utilize components of the protein secretory trafficking pathway for the direct transport of anthocyanins from ER to vacuole, and provide evidence of a novel sub-vacuolar compartment for flavonoid storage. In a subsequent work in *Arabidopsis* cells [74], the formation of AVIs strongly correlates with the specific accumulation of cyanidin 3-glucoside and derivatives, probably through the involvement of an autophagic process.

In lisianthus, it has been proposed the presence of a further type of vesicle-like bodies, finally merging in a central vacuole [67]. In this work, anthocyanin-containing pre-vacuolar compartments (PVCs) are described as cytoplasmic vesicles directly derived from ER membranes, similarly to the transport vesicles of vacuolar storage proteins. These vesicles have also been found to be filled with PAs, which are then transported to the central vacuole in *Arabidopsis* seed coat cells [48,77]. Most of these studies have shown that *Arabidopsis tt* mutants, with defects in PA accumulation, possess also important morphological alterations of the central vacuole, suggesting that the vacuole biogenesis is required for adequate PA sequestration.

In conclusion, it has been argued that the microscopy observation of these flavonoid-containing vesicles in accumulating cells could imply that the abovementioned membrane transporters are involved in flavonoid transport and storage, since these transporters may also be required for loading across any of the endomembranes involved in the trafficking. To this respect, the mechanisms proposed in different plant models could not be mutually exclusive but, on the contrary, could deliver phytochemicals in parallel to the storage compartments [17,31,50].

In addition, the model of a vesicle-mediated flavonoid transport raises also an important question on how these vesicles are firstly addressed to the correct compartment and then how they fuse to the membrane target [37]. Usually, the general mechanism of membrane trafficking requires a complex set of regulatory machinery: (i) vacuolar sorting receptor (VSR) proteins, necessary for targeted delivery of transport vesicles towards the destination compartment; (ii) soluble *N*-ethylmaleimide-sensitive factor attachment protein receptors (SNAREs), on the surface of cargo vesicles (v-SNAREs, also called R-SNARE); (iii) SNARE proteins (t-SNAREs) on target membranes, responsible for interactions with v-SNAREs, membrane fusion and cargo release; the latter are classified into Qa-SNAREs (t-SNARE heavy chains), Qb- and Qc-SNAREs (t-SNARE light chains) [78]. In plants, SNARE proteins are involved in vesicle-mediated secretion of exocytosis and endocytosis, during fundamental processes such as development, cytokinesis, primary cell wall deposition, shoot gravitropism, pathogen defence, symbiosis, abiotic stress and immune responses [79]. A direct role of these proteins in vesicular delivery of flavonoids to vacuole and/or cell wall has not yet been demonstrated, although a recent study has evidenced an involvement of secretory SNARE during extracellular release of callose and antifungal phytochemicals into the apoplast of *Arabidopsis* cells infected by powdery mildew [80].

## 6. Long Distance Transport of Flavonoids in Plants

Flavonoids may also be transported from their site of synthesis to other parts of the plant [81,82]. Flavonoids are scarcely produced in plants or organs grown in the dark, because the expression of genes encoding for CHS is strictly dependent on light [83]. Nevertheless, they are also present in roots, contributing to lateral development [84] and gravitropic response [82]. Furthermore, there is evidence on the role of flavonoids during legume nodulation [85], the induction of the hyphal branching of arbuscular mycorrhizal fungi [86], as well as the response to phosphate starvation [87] and the inhibition of polar auxin transport [88,89]. A first indication for a long distance transport has been obtained in cotyledons and flower buds of *Catharanthus roseus*, where F3′5′H is associated to phloematic tissues [83]. In *Arabidopsis* flavonoid-pathway mutants, the confocal microscopy analysis has shown that the flavonoid products accumulate inside cells and are not present in regions among cells, suggesting that the long distance movement of these molecules is symplastic [90]. By using *Arabidopsis* flavonoid-pathway mutants and *in vivo* visualization of fluorescent diphenylboric acid 2-amino ethyl ether (DBPA)-flavonoid conjugates, the same authors have demonstrated that flavonoids can be selectively transported through the plant from one organ to another [91]. These authors have inferred unidirectional movement and tissue specificity for flavonoid accumulation. This has led the authors to suggest that their distribution is mediated by an active process instead of a passive diffusion, possibly by action of a MRP/ABCC transporter [92].

## 7. Mechanism(s) of Flavonoid Transport and Regulation in Grapevine

**Figure 2:**
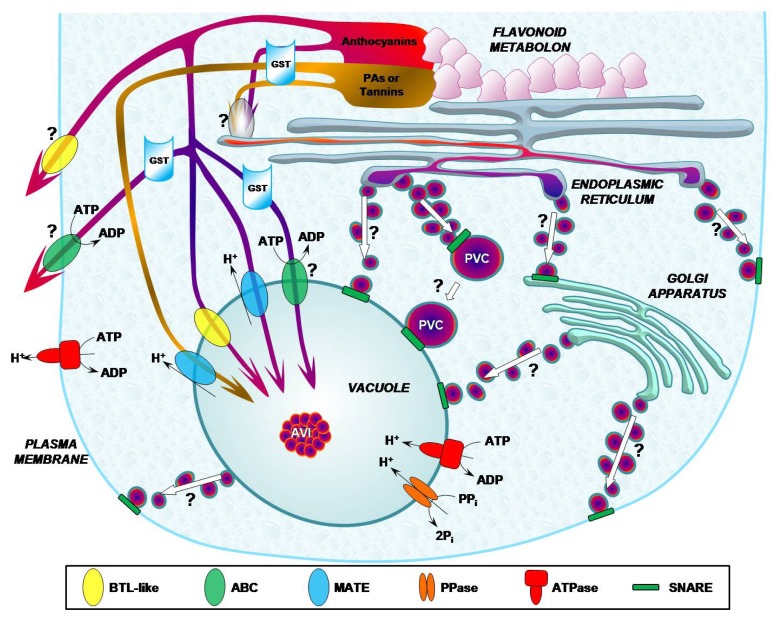
Hypothetical scheme of flavonoid transport mechanisms in grapevine cells. Fluxes of flavonoids, conjugated or not by glutathione *S*-transferases (GSTs), are shown with different colours for anthocyanins or proanthocyanidins (PAs). The main transporters, localized in tonoplast and plasma membrane, are: bilitranslocase-like protein (BTL-like); ATP-binding cassette transporters (ABC); multidrug and toxic compound extrusion transporters (MATE). Transport mediated by vesicle (multicolour circles) trafficking is indicated, as well as the main structures and proteins involved (anthocyanic vacuolar inclusions (AVI); pre-vacuolar compartments (PVC); soluble *N*-ethylmaleimide-sensitive factor attachment protein receptors (SNARE)). Question marks indicate the lack of information or hypothetical steps in the process. Flavonoid biosynthesis is shown to be localized only at the endoplasmic reticulum site; for other suggested subcellular localizations, see text in section 2.

According to previous results obtained in *Arabidopsis* and in other plant species, two different mechanisms have been also proposed in the grapevine to explain both plant flavonoid transport from the ER to the vacuole and the reverse transport from storage sites to other cell targets, where flavonoids exert their physiological effects (e.g., cell wall) [37] (Figure 2). The two distinct pathways (MVT and MTT) could contemporarily be present or, in any case, be detectable in different tissues or phenological stages in the same plant. Such behaviour confirms an old statement, interpreting flavonoid transport as a multifactorial process, involving different strategies and the contribution of several enzymes.

In spite of the great interest in this topic, direct evidence of the flavonoid transport in grapevines is scarce and most information derives from genomic and proteomic approaches. These findings initially concerned the involvement of the *GST* gene, as reported in [18] and [19]. Further experiments performed by immunochemical staining have demonstrated a localization at vacuole and vesicle level for GST [93]. The analysis of transcript profiles during berry development indicates *GST* isogenes as possible check points to evaluate fruit maturation, since they exhibit the same expression profile of anthocyanin accumulation.

Nevertheless, given the large presence of AVIs in grape berries, it could also be hypothesized the presence of different flavonoid transport systems based on vesicle trafficking. The AVI structure has been, indeed, detected in both grape cell cultures [17,94], as well in grape berry and transgenic *MYBA1*-transformed hairy roots [93]. They differ from other plant counterparts, since they have been recently described as dense organic storage structures, mainly enriched in acylated anthocyanins and long-chain PAs, appearing to be encased by a lipid membrane [13]. The MVT strategy, however, leaves an open question about the uptake of pigments into membrane compartments. This aspect plays a fundamental role, especially in flavonoid highly-enriched tissues, like in grapevine, where the large amount of these metabolites has a great physiological and technological relevance. The vesicle uploading or vacuolar transport could be accomplished by GST [16], as first demonstrated by Ageorges and co-workers [19], who identified a type I GST required for vacuolar transport of anthocyanins, and by Conn’s group [95], who characterized two anthocyanin-transporting GSTs. In addition, a study performed on several grape cultivars by a genomic approach demonstrated that GST is comprised of a narrow set of enzymes involved in anthocyanin transport [96]. It is now accepted that these enzymes, rather than by a proper GST activity, would preferably act as non-enzymatic carrier proteins (ligandins) of flavonoids, enabling their intracellular shuttling to the active transporters, such as ABC transporters responsible for trans-membrane transport. The localization of these transporters in *Vitis* has been hitherto probed at the plasma membrane [97] and, very recently, at the tonoplast [49]. In this work, it has been reported that grapevine ABCC1 is expressed in grape berry, where it mediates a GSH-dependent vacuolar transport of anthocyanidin 3-*O*-glucosides, a result suggesting a new unknown mechanism of co-transport for specific anthocyanins with free GSH.

The class of transporters involved in MTT is MATE, which has been shown to be responsible for accumulation into the grapevine vacuole of anthocyanins, particularly the acylated ones [33,93,96]. This feature could explain the high transport specificity demonstrated by MATE transporters and the presence of several isoforms [33,37,50,93]. The addition of acyl and methyl groups could be a further regulative factor, since this reaction would provide a molecular marker, which is characteristic of anthocyanins addressed to participate at AVI composition [98]. At the same time, it remains unanswered the question whether MATE is responsible for vesicle uptake of flavonoids or if it is directly involved in vacuolar transport, possibly acting as permeases [37].

Besides these two large and widespread transporter families, flavonoid accumulation could be accomplished by the activity of a putative flavonoid carrier, similar to mammalian BTL, initially found as above seen in carnation petal microsomes [54] and also identified in grapevine [99]. This membrane protein of about 30 kDa, expressed in red grape berries, is characterized by a cross-reactivity with specific antibodies raised against an epitope of rat liver BTL and mediates the active secondary transport of BSP. This transport is competitively inhibited by the anti-BTL antibody and quercetin (a flavonol present in berry), suggesting that it may transfer also flavonoids. This carrier is expressed in definite compartments, as well as during specific developmental stages of the grape berry, all peculiarities that correlate its presence with flavonoid accumulation. In fact, both immunohistochemical and immunodetection analysis have shown that BTL is mainly localized in berry skin, a known site of anthocyanin accumulation, while at subcellular level BTL expression is associated to the cell wall/plasmalemma and vacuolar compartments. These findings support the involvement of the grape BTL homologue in flavonoid accumulation inside the vacuole of tegumental cells. Such a mechanism may contribute to the formation of the AVIs by pigment precipitation that enhances the accumulation of anthocyanins and prevents their lytic degradation by vacuolar enzymes [67]. The grape BTL homologue is differently expressed during berry maturation stages in skin and pulp membranes, in both absolute quantity and expression pattern [99]. In skin tissue, the pattern of expression increases steadily from *véraison* to harvest, when it reaches a peak, following the behaviour of other proteins related to flavonoid biosynthetic pathway [19]. In pulp tissues, on the contrary, the immunodetection of the BTL homologue reveals a bell-shaped profile, with a maximum at the early ripening stage. This is an additional clue for the involvement of the protein in translocation of anthocyanin precursors and/or colourless flavonoids (e.g., PAs), which are known to be accumulated earlier with respect to anthocyanins [29]. The detection of a weak but still evident cross-reaction in vascular bundles is intriguing evidence about the participation of this carrier in long distance transport of colourless flavonoids. Indeed, Grimplet and co-workers [100] have demonstrated that the synthesis of flavonoid precursors occurs also in pulp tissues, although to a minor extent. Finally, such precursors need to be translocated into the peripheral epidermal layers for a further glycosylation and accumulation. This model shares similarity with phenylpropanoid, terpenoid and alkaloid pathways, where the intermediates, previously synthesized in the parenchyma, need to be further translocated to their final targets. This observation provides evidence for a possible role of the BTL homologue in secondary metabolite translocation inside red grape fruit [99]. A specific tissue distribution is also detectable in white berries, where the expression of BTL is, however, greater in vascular bundles than in the skin, according to the lack of anthocyanins and, consequently, of their transport to the latter tegumental tissues [101].

As above seen, the presence in plants of a long distance transport of flavonoids, mediated by vascular bundles, is also strongly suggested in grapevine by several findings concerning the physiological effects that they exert at their targets, which appear to be distinct from the synthesis site. In particular, during the ripening stage, grape berries exhibit a shift of phloem unloading from the symplastic to the apoplastic pathway, thus leading to a less efficient metabolite accumulation, due to a higher flow resistance to photo-assimilate import [102]. Hence, a cooperative activity between ATP-dependent or GST-linked primary transporters [103] and the secondary ones could be hypothesized. Therefore, late ripening stages or physiological conditions, characterized by impaired transport efficiency, seem to induce the expression of the grape BTL homologue in response to the accumulation of large amounts of flavonoids.

The existence of flavonoid transport outside the cell is generally accepted, but hitherto the only available evidence indicates the involvement of ABC transporters in this phenomenon, since neither glycosylation nor acylation of the metabolite is required [37]. In this scenario, grapevine could represent a model plant, which would be a very powerful tool to study how environmental signals influence the direction of secondary metabolite transport, and moreover, to follow *in vivo* flavonoid fluxes and the regulatory activity of different enzyme inhibitors and modulators.

Little information is available on the genetic regulation of flavonoid transport in grapevine. MYB5a and MYB5b have been demonstrated to be transcription factors regulating the grapevine general flavonoid pathway [104]. In addition, the ectopic expression of *VlMybA1-2* in grapevine is able to trigger the production and storage of anthocyanins through the activation of few genes including, besides those involved in anthocyanin synthesis, a candidate gene for antho-MATE transporter and a GST [96]. In hairy roots, it has been also shown that PA transcription factors MYBPA1 and MYBPA2 induce the ectopic expression of a MATE transporter related to *Arabidopsis* TT12 [96,105].

## 8. Involvement of Flavonoids during Stress Response in Grape

The widespread presence of flavonoids at cellular, tissue and organ level in grape, as described above, indicates that their functions are crucial for the correct development of the plant. Furthermore, flavonoids could also play a major role in plant responses to environmental cues, in particular during biotic and abiotic stresses. In this view, flavonoid synthesis, transport and allocation could be assumed as hallmarks of an adaptive metabolism, to exert protective, antibiotic and modulatory effects [106].

### 8.1. Biotic Stress

In grapevine, the stress signalling molecule methyl jasmonate (MeJA), known to be involved in biotic stress [2] has often been shown to induce an accumulation of secondary metabolites in leaves and berries, such as stilbenes (especially resveratrol and viniferin), which act as anti-microbial compounds [107]. In addition, it has been firstly reported that application of MeJA to grape cell suspension cultures, irradiated with light, increases anthocyanin production [108]. Besides, MeJA treatment, in combination with sucrose, has been studied in grapevine cell suspensions in relation to defence mechanisms. In particular, the treatment induces genes encoding pathogenesis-related (PR) proteins CHIT4c and PIN, as well as up-regulating *PAL* and *STS* genes. The latter genes are associated with a strong stilbene production. These compounds, formed starting from the general phenylpropanoid metabolism, have an anti-microbial function. Moreover, MeJA treatment determines an accumulation of *CHS* and *UFGT* genes, related to a strong increase of anthocyanins [107], and induces a hypersensitive-like response in grapevine leaves and cell suspensions, together with the accumulation of phenylpropanoid-derived compounds and defence-related products [109].

### 8.2. Abiotic Stress

#### 8.2.1. Light and UV Stress

For a long time, flavonoids have been considered only as a generic light filter to protect plant tissues from high energetic wavelengths (UV-B and UV-A). Indeed, they have been shown to protect shade-adapted chloroplast from exposure to high intensity sun flecks [110] and, in addition, can also be considered as UV-B screen, in order to protect PSII. It has been widely reported that the massive accumulation of flavonoids in external appendices is consistent with UV-screening functions in photo-protection [111]. However, recently UV-B-induced flavonoid biosynthesis does not seem to have a primary role in UV-screening [112]. Rather, UV light induces the synthesis of flavonoids with higher hydroxylation levels (dihydroxy B-ring-substituted forms, such as quercetin 3-*O* and luteolin 7-*O*-glycosides), which perform antioxidant roles, thus contributing to ROS-detoxification through chemical ROS quenching in plant cells [112].

Several studies have shown that modification of light exposure could affect flavonoid accumulation in many cultivars, such as Shiraz [111], Pinot Noir [113], Cabernet Sauvignon [114,115] and Sangiovese [116]. In these works, different methods of sunlight exclusion have been adopted, by either application of opaque boxes to bunches, as designed by Downey and co-workers [111,113,115,117], or leaf removal, and/or moving [114,116].

The expression of some flavonoid genes has been reduced by shading treatments [111,113,114,117]. In particular, the effect of light quality has been investigated [115]. Plant covering with UV-proof film does not affect proanthocyanidin amount, but this treatment remarkably decreases flavonols. Again, the transcript level of *FLS4* gene (related to flavonol biosynthesis) is lowered after shading with UV-proof film. Finally, a recent study has focused on the synergistic action between temperature and light on anthocyanin accumulation in grape berry skin [118]. It has been shown that a low temperature (15 °C) and light treatment have a positive effect on anthocyanin accumulation. It should be also underlined that the expression of different *MYB*-related genes and flavonoid-related genes are regulated independently by the two environmental factors considered [115].

#### 8.2.2. Temperature

Several studies have shown the effect of high and low temperatures on the composition or concentration of flavonoids. Low temperature has been shown to induce anthocyanin synthesis in various species [119]. In particular, Choi and co-workers [120] identified an *e*nhanced *l*evel of *a*nthocyanin *1* (*ela1*) mutant of *Arabidopsis*, which exhibits elevated levels of flavonoids and also a cold stress tolerance to a temperature of 4 °C. Effects of temperature on the content of anthocyanins in grape berry skins have been extensively studied [121–123]. Nevertheless, the mechanisms responsible for the poor coloration of berry skin at high temperatures have not been completely understood.

The effects of temperature on the biosynthesis of flavonoids and the expression levels of related genes have been examined in an *in vitro* environmental experiment, using detached grape berries [118]. This paper shows that the accumulation of anthocyanins is dependent on low temperature combined with the presence of light. Mori and co-workers [124], by a microarray analysis, have demonstrated that anthocyanin biosynthetic genes are not strongly down-regulated by high temperatures. On the contrary, the decrease in anthocyanin accumulation, under a high temperature, could result from different causes, such as accelerated anthocyanin degradation and inhibition of mRNA transcription of the anthocyanin biosynthetic genes [124].

#### 8.2.3. Water Deficit

It has been shown that plants respond to water deficit accumulating anthocyanins and other phenolics [125–127], although the metabolic inducers of such effects are still unclear [128]. The expression of anthocyanin biosynthetic genes is specifically modulated by the seasonal availability of water throughout the progress of ripening has been demonstrated [29,129]. More than 80% of the increase in anthocyanin accumulation may be correlated to a mRNA accumulation of the major anthocyanin biosynthetic genes involved in the flavonoid pathway, such as *UFGT*, *CHS* and *F3H*. Genes coding for F3′5′H and MT are also up-regulated in berries subjected to water shortage, leading to more hydroxylated and more methoxylated anthocyanin derivatives, such as malvidin and peonidin [29,129].

Previously, it has been shown [129] that anthocyanin accumulation increases after *véraison*, in either early or late water deprivation. The increase in anthocyanin accumulation results from an earlier and greater expression of the genes associated to the anthocyanin biosynthetic pathway (*F3H*, *DFR*, *UFGT* and *GST*). Manipulation of this abiotic stress, through application of moderate water deficits, can, therefore, be used as an agronomic practice, not only to modulate berry metabolite accumulation during fruit ripening, but also to change the timing of some ripening processes, since early stress determines greater effects than late stress. The onset of anthocyanin biosynthesis appears to be also anticipated. The higher anthocyanin content parallels the up-regulation of related biosynthetic genes, thus indicating that the higher concentration of anthocyanins is not merely a consequence of a higher sap concentration in fruit or of an inhibition of berry growth, but depends on an increased biosynthesis. In addition, a water shortage changes the degree of hydroxylation of anthocyanins, leading to an enrichment of purple/blue pigments, modifying grape and must colour [3]. This modification converts the pigments into moieties that are more resistant to oxidation and with a different colour.

Grimplet and co-workers [100] have also found that water deprivation induces an up-regulation of mRNA involved in several pathways of secondary metabolism. Such a phenomenon is mainly restricted to pulp and skin tissues, while seeds remain scarcely involved. These transcripts are responsible for the biosynthesis of aromatic and coloured compounds within skin and pulp tissues that ultimately impact wine quality.

Water shortage also induces an increased expression of the grape BTL homologue, in parallel with the well-known macroscopic effect on berry pigmentation [99] and the activation of the whole flavonoid biosynthetic pathway [129]. This suggests that stress conditions trigger not only the biosynthetic pathways, but also the expression of proteins involved in flavonoid transport and accumulation. Hence, such a stress seems to activate the whole metabolon involved in flavonoid metabolism, resembling the analogue phenomenon observed at *véraison* during berry development.

## 9. Conclusions

Despite the flavonoid biosynthetic pathway and its regulation mechanisms are well characterized, many aspects related to flavonoid transport and their final accumulation are still controversial. This is a crucial aspect, especially for grapevine, where large amounts of polyphenols are stored. This knowledge is also useful for understanding the allocation processes of other secondary metabolites (e.g., terpenoids and alkaloids), which are known to be synthesized in parenchymatic cells, before being translocated into and stored in other tissues. Most of the main transport models have been developed from studies in *Arabidopsis* and maize, concerning plant organs different from fruit. Nevertheless, the evidence above presented in grapevine cells suggests that flavonoids may be accumulated into the vacuole and cell wall also by a secondary active transport mediated by a protein similar to BTL. However, it is rational to argue that several pathways of flavonoid accumulation may co-exist in grape cells, as described in other plant species. Being flavonoids involved in stress phenomena, as antibiotic and modulating molecules, further studies are needed to better understand their role, particularly in relation to their transport and accumulation.

Progress in clarifying the mechanisms responsible for flavonoid transport in plant cells will be useful to manage and modify the quality and content of such metabolites in grape berry, an important economical species. This knowledge might represent a powerful tool to increase pathogen resistance in grapevine, reducing the amount of phytochemicals and, therefore, limiting environmental impact and costs of grapevine cultivation. Finally, the management of flavonoid production may also exert a positive effect on organoleptic properties of the berries, thus improving both fruit and wine quality.
